# Prevalence of syphilis and chlamydia trachomatis infection among female sex workers in Jiangsu, China: Results from a multicenter cross-sectional and venue-based study

**DOI:** 10.3389/fpubh.2022.1018724

**Published:** 2022-10-31

**Authors:** Lingen Shi, Jun Luo, Yuheng Chen, Liping Chen, Haiyang Hu, Tao Qiu, Xiaoyan Liu, Xiaoqin Xu, Yunting Chen, Zhi Zhang, Ying Zhou, Jing Lu, Gengfeng Fu

**Affiliations:** ^1^Institute for STIs and HIV Control and Prevention, Jiangsu Provincial Center for Disease Control and Prevention, Nanjing, China; ^2^Jiangsu Key Laboratory of Molecular Medicine, Medical School, Nanjing University, Nanjing, China; ^3^Department of Clinical Central Laboratory, Jiangsu Health Vocational College, Nanjing, China

**Keywords:** sexually transmitted infections, prevalence, female sex worker, HIV, China

## Abstract

**Background:**

Female sex workers (FSWs) are considered highly vulnerable to sexually transmitted infections (STIs), but available data on the prevalence of STIs among FSWs in China is limited at a provincial level. This study aimed to evaluate the prevalence of STIs and risk factors among FSWs in Jiangsu, China.

**Methods:**

We conducted a multicenter cross-sectional study in seven cities of Jiangsu to investigate the prevalence and risk factors associated with HIV and other STIs. Blood and urine were collected to test for HIV, syphilis, Hepatitis C (HCV), Neisseria gonorrhoeae (NG) and Chlamydia trachomatis (CT) infections.

**Results:**

We enrolled 3,580 FSWs. The overall prevalence of bacterial STIs was 6.2% (5.4%−7.0%). The prevalence of HIV, syphilis infection, HCV, NG and CT were 0.1% (95%CI, 0.0–0.2), 1.8% (95%CI, 1.4–2.3), 0.3% (95%CI, 0.1–0.5), 0.3% (95%CI, 0.2–0.5) and 4.3% (95%CI, 3.6–5.0), respectively. Most FSWs (85.6%) reported consistent condom use with clients in the past month. Only 10.6% of FSWs reported group sex, and 68.3% self–reported HIV testing in the previous year. According to the multivariable model, having group sex in the past year (aOR, 2.521, 95%CI: 1.366–4.651) and HIV infection (aOR, 26.260, 95%CI: 2.432–283.563) were associated with a higher risk of syphilis infection. Migrants (aOR, 1.669, 95%CI: 1.163–2.395), having a history of STIs in the past year (aOR, 4.601, 95%CI: 1.003–21.118), and NG infection (aOR, 38.549, 95%CI: 11.214–132.514) were associated with a higher risk of CT infection. On the contrary, FSWs aged older than 25 were associated with lower risk of syphilis infection (25–34: aOR, 0.339, 95%CI: 0.151–0.763) and CT infection (25–34: aOR, 0.503, 95%CI: 0.316–0.802; ≥35: aOR, 0.578, 95%CI: 0.362–0.925).

**Conclusion:**

This study's prevalence rates of syphilis and CT infections show the need to promote comprehensive STIs control and prevention strategies, including behavioral intervention and STIs screening, especially in younger high–risk populations. With the increasing coverage of HIV testing, integrating other STIs screening with HIV testing may be a reasonable way to implement comprehensive STIs control and prevention.

## Introduction

According to the World Health Organization, 374 million new infections [including Chlamydia trachomatis (CT), Neisseria gonorrhoeae (NG), Syphilis, and Trichomoniasis] were estimated nationwide in 2020. Nearly 35% of new infections were CT, one of the world's most commonly reported sexually transmitted infections (STIs) (1). The prevalence of CT and NG were high in key populations [CT: 16.3% and NG: 3.0% among men who have sex with men (MSM), CT: 17.0% and NG: 2.3% among female sex workers (FSWs)] (2, 3) than in the general population (CT: 10.2% and NG: 4.09% among women) (4). The case report of CT was under–estimated nationwide in China, considering the CT was previously not a notifiable reporting infectious diseases according to the Law of the People's Republic of China on the Prevention and Treatment of Infectious Diseases. STIs can cause serious outcomes without treatment in females, such as infertility, pelvic infection, pregnancy complications, and an increased risk of other STIs (especially HIV).

FSWs remain at high risk of HIV and other STIs worldwide. A global meta–analysis, covering 101 countries and 2,103,380 women, reported that the estimated HIV prevalence remained high and stable between 10.4 and 11.8% among FSWs from 2014 to 2018 (5). A cross–sectional study, conducted at Yunnan in 2008, reported prevalence rates of 36.8% for NG, 46.3% for CT, and 22.1% for trichomonas vaginalis among FSWs in China (6). Syphilis infection was estimated at 1.7% among FSWs in China, which was 6.8 times higher than among general population women in 2017 (7). FSWs contribute significantly to STIs transmission networks through their engagement in frequent unprotected sexual activities, multiple commercial sex partners (8–10), and limited asymptomatic STIs awareness (11). An Ecuador study reported that 25.5% of FSWs had unprotected sexual activities at least once in the previous 3 months (12). Another study in Australia observed an inconsistent condom use rate of 24% for vaginal sex with clients in 2021. About fellatio, the rate of inconsistent condom use reached 74% among clients in this population (13). In some instances, FSWs faced risks of reduced payment or intimate partner violence during negotiations for safe sex with routine clients or intimate sexual partners. Offers of higher monetary incentives for condomless sex sometimes derailed condom–use negotiations and further exposed FSWs to STIs (14, 15).

Meanwhile, a 2010 China study found that 39.8% of FSWs experienced challenges in using a condom correctly, such as condom rupture, and only used condoms during ejaculation (16). Most studies have demonstrated that STIs infection facilitates HIV infection. Controlling STIs could contribute to the Aim of “Zero AIDS” not only among FSWs but also in other high–risk sub–populations and the general population (17, 18). So, identifying the prevalence of STIs and risk characteristics of FSWs could contribute to improving strategies for controlling STIs in this population and preventing STIs from their clients and the general population.

Jiangsu, located in eastern China, belongs to the Yangtze–river economic zone, one of the three most developed and richest areas in China. The commercial sex industry flourished alongside China's rapid economic growth since the policy of economic reforms and open policies was initiated in the 1980s (19). With the rapid economic development, residents increased disposable income. Most rural–urban immigrants migrate to this province for more job opportunities and higher salaries. By 2021, the migrant population in Jiangsu reached almost 24 million, accounting for 25% of the whole population in Jiangsu province (20). Compared to indigenous residents, migrants lacked sources of health care, had poorer awareness of STIs–related knowledge, and were more likely to engage in condomless sex and commercial sex, increasing the likelihood of HIV/STIs acquisition in FSWs (21–23). Overlapping these characteristics, FSWs contribute to the worsening epidemic of HIV/STIs in Jiangsu. However, limited data on STIs surveillance among the high–risk population, especially FSWs in Jiangsu, have been reported. One study conducted in 2009 reported a high rate of syphilis (4.9%), CT (14.6%), and NG (5.4%) prevalence among FSWs in two Jiangsu province cities (24). Another study in Changzhou demonstrated high rates of CT (17.0%) and NG (2.3%) among FSWs in 2011 (3). This study aimed to investigate the prevalence of STIs and associated factors to evaluate and improve the comprehensive policies to control STIs in a provincial level.

## Methods

### Study design and participants

From April to June 2021, a cross–sectional study was conducted in seven cities, named Nanjing, Wuxi, Suzhou, Huai'an, Yancheng, Yangzhou, and Zhenjiang, in Jiangsu province. A convenient sampling approach was used to recruit eligible participants. Firstly, we mapped the sex work venues and stratified them into three subgroups, i.e., high, middle, and low titers by minimum charge (3). High–titer venues included hotels and karaoke bars, and low–titer venues included street or other public outdoor sites. Middle–titer venues included barber bars, hair salons, massage parlors, roadside shops, guesthouses, or roadside restaurants (25). Secondly, we selected the potential venues randomly according to titers. Then, we conducted an on–site survey. All eligible venue members were recruited sequentially. Based on previous studies, we restricted the proportion of participants from each titer was high (<50%), middle (>40%), and low (>10%) in each city respectively to recruit a representative sample (26, 27).

Eligible participants were biologically females aged ≥16 years and self–reported providing vaginal, oral, or anal sex for male clients within the previous 12 months.

This study was approved by Ethics Committee of Jiangsu Provincial Center for Disease Prevention and Control in Jiangsu, China. All participants provided written informed consent before this survey.

### Procedures and measures

The Jiangsu Center for Disease Control and Prevention designed a structured questionnaire for data collection. The information included socio–economic data, such as age (<25/25–34/35 and above), level of education (Secondary education or below/ High school and above), marital status (Single/married), migratory status, and monthly income (<5000/5000–7999/8000 and above, RMB). Participants who did not have official household registration in Jiangsu were deemed immigrants. Behavioral information included sexual behavior, condom use history, way to find male clients, drug history, HIV testing history, and STIs.

Based on the survey map and stratified titer of venues, the center for disease control and prevention staff contacted the house keepers or stakeholders, or managers to conduct an on–site survey. If they supported this survey, three professional staff (one for enrolling, one for screening and questionnaire, one for collecting sample of blood and urine) would take an on–site survey. Eligible participants would register their cellphone number for this survey. There was unique code linked the registration information, informed consent, questionnaire, blood and urine samples for each participant. The interviews remained anonymous throughout the survey. Each enrolled participant finished a face–to–face structured questionnaire interview.

After completing a questionnaire, each FSW donated 5 ml of venous blood with the help of trained nurses for HIV, Hepatitis C (HCV), and syphilis testing. All participants also provided 5 ml first–void urine held for more than 2 h for CT and NG testing. Finally, each participant received 50 RMB (approximately 7.5 US dollars) as participation incentives.

### Testing

The enzyme–linked immunosorbent assay (ELISA) kit screened for plasma HIV and HCV antibodies in blood samples (Zhuhai livzon Diagnostics Inc., Zhuhai, China). If the result of HIV or HCV antibodies were positive, the same blood samples were retested by another ELISA reagent (Wantai Bio–pharmacy Enterprise Co., Ltd., Beijing, China). For HCV, both tests with positive results were recorded as HCV positive. For HIV, participants need provide new blood specimens for confirmatory testing by Western Blot (MP Biomedical Asia Pacific Pte. Ltd., Singapore) if both tests with positive results. Tests with positive results from both ELISA and WB were recorded as HIV infection.

Test for treponema pallidum (TP) was conducted using ELISA (TP–ELISA, Wantai Bio–pharmacy Enterprise Co., Ltd., Beijing, China) and subsequently by toluidine red untreated serum test (TRUST, Wantai Bio–pharmacy Enterprise Co., Ltd., Beijing, China) when the antibody for TP was positive. Positive results for both TP-ELISA and TRUST were recorded as syphilis infection. If only TP–ELISA was positive, we defined as syphilis antibody positive.

Pathogens of NG and CT were tested *via* nucleic amplification tests (NAATs) using (RENDU Biotechnology Co., Ltd., Shanghai, China), followed the manufacturer's instructions. A positive NAATs result of CT or NG was defined as CT or NG infection.

If participants received any positive result of STIs, professional staff would contact with positive participants, and referred them to local infectious disease hospital for further diagnose or treatment and care.

### Statistical analysis

Descriptive analysis of categorical variables was reported as percentages. A chi–square test was used to assess the difference between subgroups. Where the chi–square test was not applicable, a fisher exact test was conducted to assess the difference between subgroups. A stepwise backward selection method was used for a multivariable logistic regression. Only variables with *P* < 0.2 were entered into the multivariable regression model. Odds ratio (OR) and adjusted Odds ratio (aOR) with a 95% Confidence interval (CI) were used to present the results. All data analyses were performed using IBM SPSS STATISTISCS (version 19.0, SPSS Inc., Chicago, IL, USA).

## Results

### Socio–demographic characteristics of FSWs

In total, we enrolled 3,580 FSWs from seven cities of Jiangsu province, including 985 (27.5%) from low titer venues, 1,866 (52.1%) from middle titer venues, and 729 (20.4%) from high titer venues. The mean age of FSWs was 35.0 years old (range, 16.0–65.0 years). The majority of FSWs were older than 35 years old (47.1%), with secondary education or below (69.9%), and currently married (72.4%). Among FSW participants, 43.5% were migrants, and 51.6% earned < 5000 RMB monthly (Table 1).

**Table 1:** Socio–demographic characteristics of 3,580 FSWs in a multi–center cross–sectional and venue–based study in Jiangsu, China.

**Variables**	**Number**	**Percentage (%)**
**Age** (Mean, Range)	35.0 (16.0–65.0)	
<25	487	13.6
25–34	1408	39.3
≥35	1685	47.1
**Education level**		
Secondary education or below	2503	69.9
High school and above	1077	30.1
**Marital status**		
Single	988	27.6
Currently married	2592	72.4
**Migrant**		
Yes	1557	43.5
No	2023	56.5
**Monthly income (CNY)**		
<5000	1848	51.6
5000–7999	931	26.0
≥8000	801	22.4
**Recruitment tier**		
Low	985	27.5
Middle	1866	52.1
High	729	20.4

### STIs–related behavioral characteristics of FSWs

Most FSWs (82.6%) reported staying in the enrolled cities for commercial sex work for more than 6 months, and 43.1% of FSWs stayed in the same city for their last venue for commercial sex work (Table 2).

**Table 2:** Risk factors for any sexual transmitted infections (STIs) among FSWs in Jiangsu, China.

**Variables**	**Number**	**Percentage (%)**
**Duration time of commercial sex work in enroll city**		
<6 months	624	17.4
≥6 months	2956	82.6
**The last venue for selling sex**		
Other provinces	755	21.1
Other cities in Jiangsu Province	861	24.1
Same city	1543	43.1
None	421	11.8
**Condom use with the last client**		
Yes	3459	96.6
No	121	3.4
**Have unprotected sex with a customer in the past month**		
Yes	515	14.4
No	3065	85.6
**Group sex in the past year**		
Yes	379	10.6
No	3201	89.4
**Find clients in fixed venues**		
Yes	3124	87.3
No	456	12.7
**Find clients by phone**		
Yes	1105	30.9
No	2475	69.1
**Find clients by internet**		
Yes	1692	47.3
No	1888	52.7
**The main way to find clients**		
Fixed Venues	2248	62.8
Telephone reservation	291	8.1
Internet	1041	29.1
**Ever used illicit drugs in the past year**		
Yes	8	0.2
No	3572	99.8
**History of STIs in the past year**		
Yes	13	0.4
No	3567	99.6
**History of STIs symptoms in the past year**		
Yes	187	5.2
No	3393	94.8
**HIV testing in the past year**		
Yes	2445	68.3
No	1135	31.7

Considering the way to find clients, 87.3% of FSWs reported ever in local fixed venues. 30.9% of FSWs found clients using the phone and providing door–to–door service, and 47.3% found clients using the internet. The main way to find clients was fixed venues (62.8%) and the internet (29.1%) (Table 2).

Most FSWs (85.6%) reported consistent condom use with clients in the past month. When they reached the last episode of commercial sex, 96.6% of them reported condom use. On the contrary, 14.4% of FSWs reported unprotected sex with clients in the past month, and 10.6% reported group sex in the previous year. Only 8 (0.2%) of FSWs used illicit drugs in the past year (Table 2).

### Testing for HIV and other STIs

Overall, 2,445 (68.3%) FSWs reported receiving HIV testing the previous year, 187 (5.2%) admitted having symptoms of STIs, and 13 (0.4%) reported STIs diagnosis in the previous year (Table 2).

### Prevalence of HIV and other STIs

In total 3,580 (100.0%) blood specimens and 3,307 (92.4%) urinary specimens were collected. Only 4 (0.1%, 95%CI, 0.0%−0.2%) case of HIV infection was found. The overall prevalence of bacterial STIs (syphilis, CT or NG) was 6.2% (95%CI, 5.4%−7.0%). The prevalence of syphilis antibody positive was 4.9% (95%CI, 4.2–5.5), and the prevalence of current syphilis infection was 1.8% (95%CI, 1.4–2.3). The prevalence of single CT and NG infections was 4.3% (95%CI, 3.6–5.0) and 0.3% (95%CI, 0.2–0.5), respectively. Only one FSW with current syphilis infection was co–infected with HIV. 4.3% (6/141) of FSWs with CT were co–infected with NG (Table 2), and 11 (0.3%, 95%CI, 0.1–0.5) cases of HCV infections were found (Table 3).

**Table 3:** STIs prevalence among FSWs in Jiangsu, China.

**Variables**	**Number**	**Percentage (%)**
**HIV infection**		
Yes	4	0.1
No	3576	99.9
**HCV infection**		
Yes	11	0.3
No	3569	99.7
**Syphilis antibody positive**		
Yes	174	4.9
No	3406	95.1
**Syphilis infection**		
Yes	65	1.8
No	3515	98.2
**Co–infection (HIV+Syphilis)**		
Yes	1	0.03
No	3579	99.97
**Total bacterial STIs infection (Syphilis/CT/NG)**		
Yes	221	6.2
No	3359	93.8
**NG infection (*****N*** **=** **3307)**		
Yes	11	0.3
No	3296	99.7
**CT infection (*****N*** **=** **3307)**		
Yes	141	4.3
No	3166	95.7
**Co–infection (CT+NG) (N** **=** **141)**		
Yes	6	4.3
No	135	95.7

STIs, Sexually transmitted infections; HCV, Hepatitis C; CT, Chlamydia trachomatis; NG, Neisseria gonorrhoeae.

### Factors associated with syphilis and CT infection

Chi–square or Fisher exact tests were used to compare syphilis and CT infection differences between subgroups of variables. Syphilis infection was significantly associated with age, migrant status, monthly income, group sex in the past year (all *P* < 0.05). CT infection was significantly associated age, marital status, migrant status, recruitment titer, and NG infection (all *P* < 0.05) (Table 4).

**Table 4:** Differences in syphilis and CT infection between subgroups of variables among FSWs in Jiangsu, China.

**Variables**	**Syphilis infection (*****N*** = **3580)**			**CT infection (*****N*** = **3307)**		
	**No. positive (%)**	**No. negative (%)**	***P*–value**	**No. positive (%)**	**No. negative (%)**	***P*–value**
**Age group (years)**			0.006			0.008
<25	12 (18.5)	475 (13.5)		32 (22.7)	427 (13.5)	
25–34	13 (20.0)	1395 (39.7)		50 (35.5)	1234 (39.0)	
≥35	40 (61.5)	1645 (46.8)		59 (41.8)	1505 (47.5)	
**Education**			0.486			0.451
Secondary education or below	48 (73.8)	2455 (69.8)		95 (67.4)	2227 (70.3)	
High school and above	17 (26.2)	1060 (30.2)		46 (32.6)	939 (29.7)	
**Marital status**			0.793			0.037
Single	17 (26.2)	971 (27.6)		49 (34.8)	848 (26.8)	
Currently married	48 (73.8)	2544 (72.4)		92 (65.2)	2318 (73.2)	
**Migrant**			0.002			0.009
Yes	16 (24.6)	1541 (43.8)		76 (53.9)	1354 (42.8)	
No	49 (75.4)	1974 (56.2)		65 (46.1)	1812 (57.2)	
**Monthly income (CNY)**			0.040			0.897
<5000	40 (61.5)	1808 (51.4)		72 (51.1)	1680 (53.1)	
5000–7999	8 (12.3)	92 3(26.3)		39 (27.7)	838 (26.5)	
≥8000	17 (26.2)	784 (22.3)		30 (21.3)	648 (20.5)	
**Recruitment tier**			0.164			0.028
Low	24 (36.9)	961 (27.3)		30 (21.3)	889 (28.1)	
Middle	32 (49.2)	1834 (52.2)		90 (63.8)	1658 (52.4)	
High	9 (13.8)	720 (20.5)		21 (14.9)	619 (19.6)	
**Duration time of commercial sex work in enroll city**			0.123			0.766
<6 months	16 (24.6)	2907 (82.7)		25 (17.7)	531 (16.8)	
≥6 months	49 (75.4)	608 (17.3)		116 (82.3)	2635 (83.2)	
**The last venue for selling sex**			0.272			0.676
Other provinces	8 (12.3)	747 (21.3)		28 (19.9)	665 (21.0)	
Other cities in Jiangsu Province	20 (30.8)	841 (23.9)		34 (24.1)	784 (24.8)	
Same city	28 (43.1)	1515 (43.1)		57 (40.4)	1333 (42.1)	
None	9 (13.8)	412 (11.7)		22 (15.6)	384 (12.1)	
**Condom use with the last client**			0.891			0.584^*^
Yes	63 (96.9)	3396 (96.6)		137 (97.2)	3071 (97.0)	
No	2 (3.1)	119 (3.4)		4 (2.8)	95 (3.0)	
**Have unprotected sex with a customer in the past month**			0.556			0.999
Yes	11 (16.9)	504 (14.3)		20 (14.2)	449 (14.2)	
No	54 (83.1)	3011 (85.7)		121 (85.8)	2717 (85.8)	
**Group sex in the past year**			0.001			0.668
Yes	15 (23.1)	364 (10.4)		14 (9.9)	351 (11.1)	
No	50 (76.9)	3151 (89.6)		127 (90.1)	2815 (88.9)	
**The main way to find clients**			0.242			0.418
Fixed Venues	43 (66.2)	1027 (29.2)		92 (65.2)	963 (30.4)	
Telephone reservation	8 (12.3)	283 (8.1)		8 (5.7)	269 (8.5)	
Internet	14 (21.5)	2205 (62.7)		41 (29.1)	1934 (61.1)	
**Ever used illicit drugs in the past year**			0.864^*^			0.295^*^
Yes	0 (0.0)	8 (0.2)		1 (0.7)	7 (0.2)	
No	65 (100.0)	3507 (99.8)		140 (99.3)	3159 (99.8)	
**HIV testing in the last year**			0.708			0.168
Yes	43 (66.2)	2402 (68.3)		91 (64.5)	2216 (70.0)	
No	22 (33.8)	1113 (31.7)		50 (35.5)	950 (30.0)	
**History of STIs in the past year**			0.623			0.077^*^
Yes	0 (0.0)	13 (0.4)		2 (1.4)	9 (0.3)	
No	65 (100.0)	3502 (99.6)		139 (98.6)	3157 (99.7)	
**History of STIs symptoms in the past year**			0.432			0.388
Yes	2 (3.1)	185 (5.3)		10 (7.7)	171 (5.4)	
No	63 (96.9)	3330 (94.7)		131 (92.9)	2995 (94.6)	
**HIV infection**			0.071^*^			0.840^*^
Yes	1 (1.5)	3 (0.1)		0 (0.0)	4 (0.1)	
No	64 (98.5)	3512 (99.9)		141 (100.0)	3162 (99.9)	
**HCV infection**			0.817^*^			0.381
Yes	0 (0.0)	11 (0.3)		1 (0.7)	10 (0.3)	
No	65 (100.0)	3504 (99.7)		140 (99.3)	3156 (99.7)	
**Syphilis infection**						0.525^*^
Yes	–	–		2 (1.4)	58 (1.8)	
No	–	–		139 (98.6)	3108 (98.2)	
**CT infection**			0.524^*^			
Yes	2 (3.1)	139 (4.0)		–	–	
No	63 (96.9)	3376 (96.0)		–	–	
**NG infection**			0.183^*^			<0.001^*^
Yes	1 (1.5)	10 (0.3)		6 (4.3)	5 (0.2)	
No	64 (98.5)	3505 (99.7)		135 (95.7)	3161 (99.8)	

STIs, Sexually transmitted infections; HCV, Hepatitis C; CT, Chlamydia trachomatis; NG, Neisseria gonorrhoeae;

* Fisher exact test; –, Not available.

In the multivariable model, FSWs who were aged among 25–34 (aOR, 0.339, 95%CI: 0.151–0.763), migrants (aOR, 0.466, 95%CI: 0.257–0.845), and monthly income of 5000–7999 (aOR, 0.450, 95%CI: 0.205–0.991) were less likely to have syphilis infection. On the contrary, having group sex in the past year (aOR, 2.521, 95%CI: 1.366–4.651) and HIV infection (aOR, 26.260, 95%CI: 2.432–283.563) were associated with a higher risk of syphilis infection. FSWs who were migrants (aOR, 1.669, 95%CI: 1.163–2.395), having a history of STIs in the past year (aOR, 4.601, 95%CI: 1.003–21.118), and NG infection (aOR, 38.549, 95%CI: 11.214–132.514) were more likely to have CT infection (Table 5).

**Table 5:** Factors independently associated with syphilis and CT infection among FSWs in Jiangsu, China.

**Variables**	**Syphilis infection**		**CT infection**	
	**OR (95%CI)**	***P*–value**	**OR (95%CI)**	***P*–value**
**Age group (years)**				
<25	Reference		Reference	
25–34	0.339 (0.151–0.763)	0.009	0.503 (0.316–0.802)	0.004
≥35	0.749 (0.355–1.579)	0.447	0.578 (0.362–0.925)	0.002
**Migrant**				
Yes	0.466 (0.257–0.845)	0.012	1.669 (1.163–2.395)	0.057
No	Reference		Reference	
**Month income (CNY)**				
<5000	Reference			
5000–7999	0.450 (0.205–0.991)	0.047		
≥8000	1.461 (0.749–2.848)	0.266		
**Group sex in the past year**				
Yes	2.521 (1.366–4.651)	0.003		
No	Reference			
**HIV infection**				
Yes	26.260 (2.432–283.563)	0.007		
No	Reference			
**History of STIs in the past year**				
Yes			4.601 (1.003–21.118)	0.050
No			Reference	
**NG infection**				
Yes			38.549 (11.214–132.514)	<0.001
No			Reference	

STIs, Sexually transmitted infections; CT, Chlamydia trachomatis; NG, Neisseria gonorrhoeae.

## Discussion

To our knowledge, this study was a large sample size and recruited from various categories of sex venues among FSWs in Jiangsu, China. In this multicenter cross–sectional study, the total prevalence of bacterial STIs was 6.2% for FSWs. The STIs prevalence among FSWs were 0.1% for HIV, 1.8% for syphilis infection, 0.3% for HCV, 4.3% for CT, and 0.3% for NG, respectively. Nearly half of FSWs were migrants from other provinces, and almost 100% of FSWs reported condom use with their last client. However, almost one–sixth of FSWs reported unprotective sex with clients in the past month. One in ten FSWs reported group sex in the past year, and 68.3% of FSWs got HIV testing in the past year. In the multiple logistic regression model, having group sex and HIV infection were risk factors for syphilis infection; being a migrant, having a history of STIs, and previous NG infection increased the risks of CT infection.

In our study, the CT and NG prevalence was 4.3 and 0.3%, respectively, lower than previous CT and NG prevalence rates reported among FSWs in Jiangsu by other studies. For instance, one study reported a CT prevalence of 14.61% and an NG prevalence of 5.42% in 2009 (24). Another study found a CT prevalence of 17.0% and an NG prevalence of 2.3% among FSWs in 2011 (3). It is possible that different specimens contribute to variations in sensitivity for CT/NG testing may explain the difference between our findings and other study findings (28). Guo and Tang collected cervical specimens to test CT and NG infection using NAATs in their studies. Our study collected first–void urine specimens to test CT and NG using NAATs. Also, compared with 40.7% of FSWs who reported consistent condom use with clients in the last month in Guo's study, 85.6% of participants reported consistent condom use in our study, which might imply a higher rate of STIs prevention among our sample. Nonetheless, the difference in reported prevalence rates implies that multiple specimens could be collected from different anatomical sites to evaluate the actual prevalence of CT/NG among FSWs in future studies.

Our result called for increased attention to stop STIs transmission among youth who sell sex. We found a high prevalence of CT among FSWs, particularly in <25 years old participants. Young–aged FSWs accounted for nearly one to five FSWs, suggesting a significant burden of bacterial STIs (syphilis and CT infection) compared with other age groups in our study. This finding corroborated with results from previous studies (11, 25, 29). Meanwhile, the high rate of CT infections indicated continuing high levels of risky behaviors among FSWs in Jiangsu. In China, there was still no national surveillance plan for CT and NG among the high–risk population. Our study's observed high rate of bacterial STIs infection also underlines the impendence and necessity of implementing bacterial STIs interventions (especially CT and NG) among FSWs.

Condom use can reduce HIV or other STIs transmission at a community level (30, 31). Since 2004, Jiangsu has promoted 100% condom use among FSWs to prevent HIV and other STIs. In our study, nearly 100% of FSWs reported condom use during their last sexual episode, which was higher than the country–level rate of condom use (77%) among FSWs (32). Nine to ten FSWs reported consistent condom use with their clients in the past month, which was higher than the rate (50.5%) in Zhejiang, another province in the Yangtze–river economic zone (33). Since 2010, the HIV prevalence consistently remained below 0.5%, and the syphilis infection prevalence also decreased from 5.32 to 1.72% from 2011 to 2020 among FSWs (unpublish data). The sentinel surveillance showed that condom promotion could effectively prevent HIV and syphilis transmission among the FSWs sub–population.

In this study, we found that 68.3% of FSWs received HIV testing last year, which was higher than the result from a meta–analysis conducted using data from China (34). China adopted regular HIV testing every three to six months among the high–risk population as part of its national programming (35). With this national guidance, 76.7% of FSWs reported taking at least one HIV test in the last year, from 2018 to 2019, in nine cities in China (36). This study's higher rate of HIV testing may be due to comprehensive prevention measures, such as condom promotion, educational campaigns, and HIV testing and counseling implemented over the years (37, 38). However, there were no recommended CT/NG screening strategies in China, even though a high prevalence of CT/NG was observed in multiple studies among the high–risk population in China (39–41). The increasing trend of HIV testing implies that integrating CT/NG testing with HIV testing was a reasonable way to screen for STIs, considering nearly 50% of NG and 75% of CT remain asymptomatic among FSWs (11, 42).

This study is the first–time urine samples have been collected for CT and NG testing in a community in Jiangsu. Based on clinical study protocols, previous studies usually collected cervical specimens to test CT and NG. However, according to a study on model sampling for HPV–based cervical cancer screening in 2019, 79% of participants had positive feelings about urine–based testing rather than cervical–vaginal sampling (18%) (43). According to findings from two studies conducted in Jiangsu, the rate of providing a cervical specimen for CT and NG tests was 28.57% (24) and 71.59% (3), respectively. However, 92.37% of participants in our study provided a urine sample for the CT/NG test, which showed high acceptability and feasibility of using urine samples. Furthermore, Cervical–based sampling needs a professional clinician and appropriate space for the sampling. In a community–based survey, we could not provide enough room for cervical samples, or participants refused to provide cervical swabs. Therefore, using first–void urine samples was more convenient for both researchers and participants. Further study on the sensitivity of CT/NG from different anatomical sites should be undertaken.

## Limitations

Several limitations of this study should be considered for interpreting the results. First, as a hidden and marginalized group, we could not reach this population without the cooperation of stakeholders or venue owners, or managers in potential venues. Participants were not randomly recruited based on a convenient sample in this study. Sample representativeness might be jeopardized by the venue–based sampling method, which overlapped with the increasing proportion of non–venue–based venue–seeking sex activities. Second, we only used the urine samples to test NG and CT among FSWs, not rectal or pharyngeal samples. Even though the rate of urogenital chlamydia was higher than that of anorectal chlamydia, the prevalence of CT might be underestimated without a multiple anatomical site specimens (44). Nearly 10% of participants refused to collect a urine sample for the NG and CT testing. That also might have contributed to an underestimation of the prevalence of NG and CT. Third, recall bias could not be ignored since all data associated with risk behaviors are based on self–report. Finally, we could not ignore the impact of COVID−19 pandemic on the prevalence of STIs among FSW. Regardless, our study has provided representative data to show the current rates of STIs prevalence and associated factors related to STIs infection among FSWs in Jiangsu, which may be used to modify future prevention strategies.

## Conclusions

The observed percentages of syphilis and CT infections show the need to promote more comprehensive STIs control and prevention strategies, including behavioral intervention and STIs screening, especially among younger high–risk populations. The increasing coverage of HIV testing implies that integrating other STIs screening with HIV testing may be a reasonable way to implement comprehensive STIs control and prevention.

## Data availability statement

The raw data supporting the conclusions of this article will be made available by the authors, without undue reservation.

## Ethics statement

The studies involving human participants were reviewed and approved by Ethics Committee of Jiangsu Provincial Center for Disease Prevention and Control. The patients/participants provided their written informed consent to participate in this study.

## Author contributions

LS, JuL, and GF had the original idea. LC, HH, XX, ZZ, YZ, and JiL conducted the testing for STIs. YuhC, TQ, XL, and YunC collected the data. LS and JuL analyzed the data. LS wrote the main manuscript text. All authors contributed to manuscript revision and approved the submitted version.

## Conflict of interest

The authors declare that the research was conducted in the absence of any commercial or financial relationships that could be construed as a potential conflict of interest.

## Publisher's note

All claims expressed in this article are solely those of the authors and do not necessarily represent those of their affiliated organizations, or those of the publisher, the editors and the reviewers. Any product that may be evaluated in this article, or claim that may be made by its manufacturer, is not guaranteed or endorsed by the publisher.

## Abbreviations

CT: Chlamydia trachomatis
NG: Neisseria gonorrhoeae
STIs: Sexually transmitted infections
FSWs: Female sex workers
ELISA: Enzyme-linked immunosorbent assay
TP: Treponema pallidum
TRUST: Toluidine red untreated serum test
NAATs: Nucleic amplification tests
OR: Odds ratio
aOR: adjust Odds ratio
CI: Confidence interval.
